# eHealth educational intervention: effects of an online video program in prevention of the pelvic women’s health—a non-randomized experimental study

**DOI:** 10.1007/s00404-025-08200-6

**Published:** 2025-10-03

**Authors:** Laura Fuentes-Aparicio, Fernando Domínguez-Navarro, Ana Maudos-Soriano, David Hernández-Guillén

**Affiliations:** 1 Physiotherapy in Motion, Multi Speciality Research Group (PTinMOTION), Department of Physiotherapy, University of Valencia, Carrer de Gascó Oliag, 5, 46010 Valencia, Spain; 2 Department of Physiotherapy, Faculty of Physiotherapy, University of Valencia, Carrer de Gascó Oliag, 5, 46010 Valencia, Spain

**Keywords:** eHealth, Pelvic floor disorders, Knowledge, Women, Education

## Abstract

**Purpose:**

This study aimed to evaluate the effects of an online educational video program on pelvic floor health knowledge, sexual function, quality of life, and motivation for physical activity in women engaged in low- and high-intensity exercise.

**Methods:**

A two-arm clinical trial was conducted with 60 women aged 18–35, divided into low- and high-intensity activity groups. Participants completed a six-week online video course covering pelvic floor anatomy, function, and self-management. Pre- and post-intervention assessments included validated questionnaires: PIKQ (pelvic floor knowledge), FSFI-19 (sexual function), BREQ-2 (exercise motivation), and EQOL-6D (quality of life). Data were analyzed using repeated measures ANOVA.

**Results:**

Pelvic floor knowledge improved significantly in both groups (low-intensity: *p* < 0.001, *d* = 0.668; high-intensity: *p* < 0.001, *d* = 0.825), especially in POP-related knowledge (*p* < 0.001). Sexual function improved in desire (*p* = 0.046) and arousal (*p* = 0.027) for the low-intensity group, and in pain during intercourse for the high-intensity group (*p* = 0.049). No significant changes were found in exercise motivation (*p* > 0.05). Anxiety and depression scores improved only in the low-intensity group (*p* = 0.031).

**Conclusions:**

The online program effectively enhanced pelvic floor knowledge and aspects of sexual function, showing promise as a preventive educational tool for active women.

**Trial registration:**

Registered in ClinicalTrials.gov (NCT05667012). Last update: 2024-12-06.

## What does this study add to the clinical work

**Table:** 

An online video-based educational intervention can improve sexual function and quality of life in women, regardless of their physical activity level. Its accessible and low-cost format makes it a promising tool, although further studies are needed to optimize its impact on pelvic floor knowledge.	

## Introduction

Urinary incontinence (UI) and pelvic organ prolapses (POP) are among the most prevalent pelvic floor disorders in physically active women [1]. Those conditions are widespread among young female athletes, particularly those around 20 years old [2], and can lead to reduced physical capacity, sexual dysfunction, and a negative impact on quality of life [3].

Elevated abdominal pressures exerted during physical activities may predispose women to pelvic floor dysfunction [4], with some studies suggesting a relationship between the intensity of training and the severity of pelvic floor dysfunction, including UI and POP [5], due to the degree of pelvic floor impact induced during exercise. Several lines of evidence suggest that high-impact physical exercise is an important risk factor for the development of urinary incontinence symptoms [6, 7]. For instance, da Silva et al. [8] observed a six times higher prevalence ratio of UI symptoms among those engaged in CrossFit compared to sedentary women. Similarly, it has been found that in female runners, the rates are higher in those who train at greater intensity and frequency [9].

Although the therapeutic management of these conditions is well-established [10], evidence-based, and supported by scientific consensus [10, 11], its implementation remains limited, primarily due to a lack of awareness among women who are affected or at risk of UI and POP [12]. This includes insufficient knowledge about the pelvic floor’s anatomy and function, poor recognition of early warning signs, and difficulties in correctly activating pelvic floor muscles [13, 14]. The need for greater health education regarding pelvic floor management is evident across different age groups and conditions, including among women who participate in sports. Despite an expressed interest in receiving better education on the topic, this knowledge gap persists [15]. Several authors have pointed out that gender stereotypes in sports have led to a lower prioritization of research focused on female athletes. This imbalance has contributed to reduced professionalization and limited scientific and clinical development in women’s sports. As a result, preventive programs targeting conditions that predominantly affect female athletes—such as those related to the pelvic floor—remain underdeveloped and insufficiently implemented [16].

Proposed educational programs for pelvic floor management include face-to-face sessions, group classes, or internet-based interventions. Overall, these strategies have shown beneficial effects on symptom incidence, severity, self-efficacy, and perceived health status for women diagnosed with UI and POP [17–19]. Additionally, online video-based interventions are considered feasible and easily applicable, taking advantage of technological advancements and the widespread use of communication technologies [20]. While such video-based educational interventions have been used for pelvic floor-related health issues, such as chronic pain management and rehabilitation [21], their impact on the prevention of UI and POP in active women remains understudied. Hence, their suitability for female athletes—those at higher risk of developing these conditions—is still unclear. Additionally, whether women engaged in more intense activity (more likely to suffer these conditions) will benefit more than those in low-intensity activity (less likely) also remains unknown.

Therefore, this study aims to analyze the effects of an online educational program on pelvic floor health knowledge among women participating in low- and high intensity training, and to compare these effects. Concretely, the effects were analyzed in terms of sexual function, quality of life, physical activity, and satisfaction. It was hypothesized that this intervention would provide enhanced outcomes in certain parameters, in both training groups, in a similar manner.

## Methods

### Study design

The present study is a non-randomized experimental design. Participants were classified into two groups (low vs. high physical activity) according to their level of physical activity, assessed with the International Physical Activity Questionnaire–Short Form (IPAQ-SF). Both groups received the same intervention, consisting of an online educational course. The same outcomes were analyzed in both groups, and pre- to post-intervention changes were compared to evaluate differential effects between groups. This research was registered in ClinicalTrials.gov (NCT05667012) and was approved by the Ethics Committee of Universitat de Valencia (code: 2432372, 12-1-2023).

### Subjects and recruitment

To provide an educational intervention to prevent pelvic floor injuries, women without a current diagnosis of pelvic floor dysfunction were contacted. The inclusion criteria were as follows: (I) women aged between 18 and 35 years old, (II) engaged at a professional or semi-professional level in a sport or physical activity, (III) able to understand the Spanish language. Additionally, the exclusion criteria were: (I) to have undergone any pelvic floor dysfunction or any gynecological surgical intervention process, (II) being pregnant, or having given birth in the last 6 months, (III) having suffered or currently suffering from respiratory, musculoskeletal, metabolic, or neurological diseases, and (IV) having been involved previously in an educational program for health-related conditions. Moreover, those who refused to participate or failed to complete the questionnaires were also excluded.

The study was disseminated through advertising posters (in both poster and PDF formats) and distributed via word of mouth and electronic communication channels. Potential candidates were evaluated for inclusion by the principal investigator. Those who met the criteria were informed verbally and in writing about the nature, purpose, and requirements of the research, and signed the informed consent once they agreed to participate, following the guidelines of the Declaration of Helsinki and its subsequent updates.

### Sample size

No previous studies conducting an online program health course in physically active women were identified to guide a power analysis for our study. The G*power software (version 3.1.9.7) was used to calculate the sample size. For the primary outcome—*Prolapse and Incontinence Knowledge Questionnaire (PIKQ),* a margin of error of 0.05 with a 95% confidence interval was used, with a power of 0.8, and a sample proportion of 50%. Assuming a 20% loss, a total of 66 participants would be required to be recruited across both groups.

### Group categorization

Participants were divided into two groups based on the amount and type of physical activity regularly performed (low and high intensity), according to the results of the validated IPAQ-SF [22]. This questionnaire was administered in person to all participants before the beginning of the intervention. Participants with results indicating they were engaged in high-intensity or low-intensity physical activity with a frequency of at least three thousand metabolic equivalent (MET) minutes per week, or approximately four hours per week of intense or vigorous exercise, were categorized into the high-intensity group. Conversely, those with results below that level were allocated to the low-intensity group.

### Intervention

**Fig. 1:**
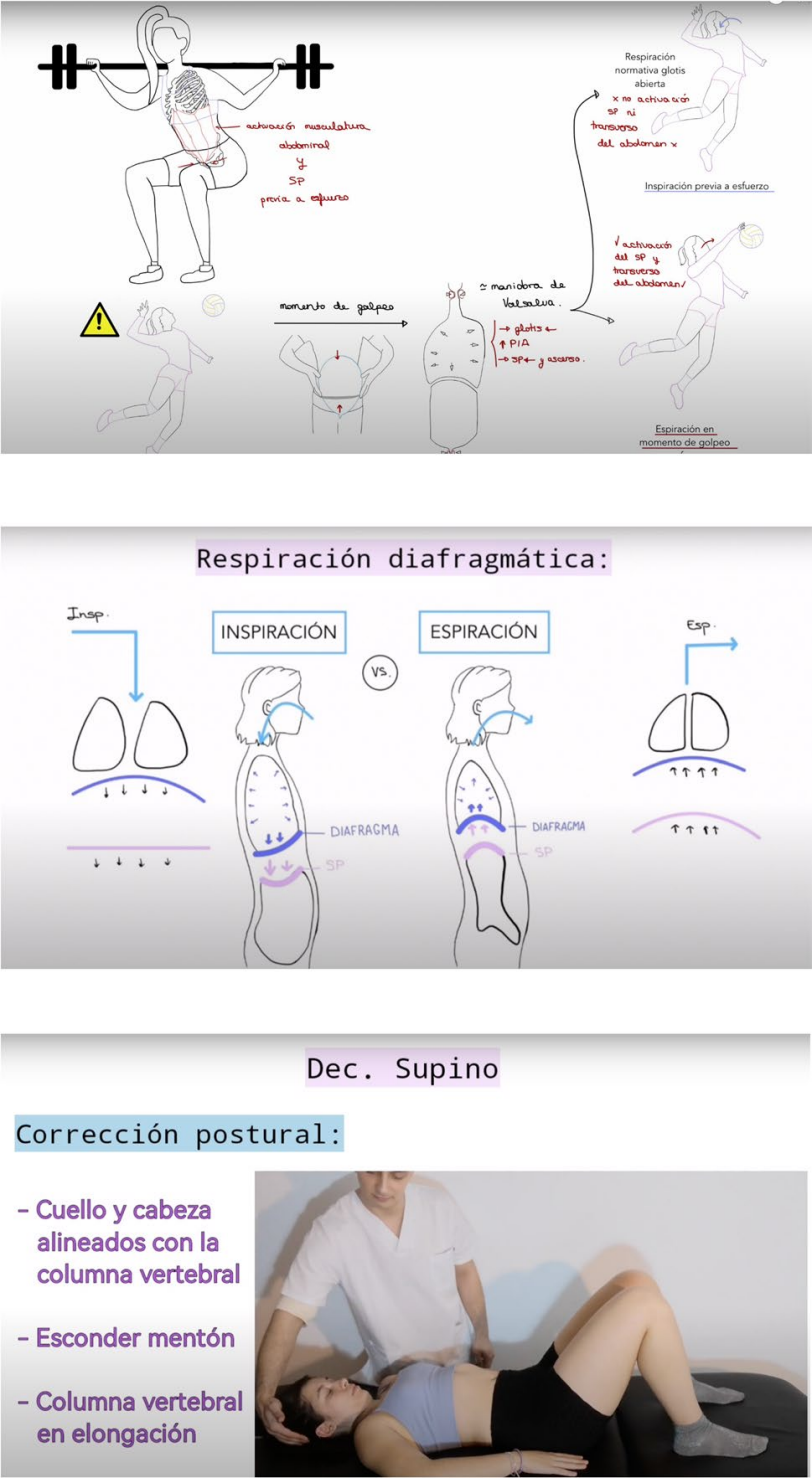
Examples of image clips from the explanatory videos

All participants received an eHealth educational intervention comprising six online videos on pelvic floor anatomy, function, and self-management. The program aimed to improve knowledge and confidence in pelvic health, using illustrated and animated visuals with voiceover narration (see Fig. 1).

Each video lasted 10–15 min and followed a progressive structure—from basic anatomy and function to practical self-care and prevention strategies for pelvic floor disorders. Content was grounded in current scientific evidence to ensure accuracy and alignment with best practices. The videos were designed to be clear, engaging, and accessible to enhance understanding and retention. Full video details, including titles, summaries, and access links, are available in Appendix 1.

Videos were uploaded weekly to YouTube with private access. Participants received access codes via text and were asked to confirm viewing. If a video was not watched within four days, a reminder was sent.

### Outcome’s assessment

This study used a test–retest design to evaluate the intervention’s effects. Participants completed an online survey via LimeSurvey.org (licensed by the University of Valencia), accessed through email or text. If not completed within two weeks, a research team member sent a reminder. The survey was administered before and after the online course, following the viewing of six videos.

The survey included four validated, self-administered Spanish questionnaires, with contact information provided for support. Knowledge regarding pelvic floor disorders and their management is an essential component in promoting adherence to preventive and therapeutic strategies. Therefore, the Prolapse and Incontinence Knowledge Questionnaire was selected as the primary outcome, given its direct alignment with both the intervention and the objective of the study. To obtain the impact of the intervention, secondary outcomes were also considered, including sexual function, exercise behavior, and general health, which were assessed using the Female Sexual Function Index, the Behavioral Regulation in Exercise Questionnaire-2, and the European Quality of Life-5 Dimensions, respectively.

*Prolapse and incontinence knowledge questionnaire (PIKQ)*: Contains 24 items (12 on urinary incontinence, 12 on pelvic organ prolapse) assessing knowledge of etiology, diagnosis, and treatment. Responses: “Agree,” “Disagree,” or “Uncertain” (one correct answer). Scores range from 0–12 per section. Internal consistency (CFI): 0.81–0.91 [24].

*Female sexual function index (FSFI-19):* 19 items across six domains (desire, arousal, lubrication, orgasm, satisfaction, pain), each with five response options. Total score ranges from 2 (minimal) to 36 (maximum). This questionnaire provides information on the participants’ sexual health and has shown an excellent intraclass correlation coefficient (ICC = 0.90–0.94) [23].

*Behavioral regulation in exercise questionnaire-2 (BREQ-2):* 19 items measuring motivation types toward exercise, based on Self-Determination Theory (intrinsic, integrated, identified, introjected, and external motivation). The responses range from “Not at all true” to “Very true”. This tool has demonstrated good reliability, with an internal consistency (CFI) of 0.92 [25].

*European quality of life-5 dimensions (EQOL-5D):* 5 items assessing mobility, self-care, daily activities, pain, anxiety/depression, and overall health. Excellent reliability (ICC = 0.91) [26].

### Data management and analysis

Survey responses were exported from LimeSurvey into data tables, combining pre- and post-intervention data and categorizing participants by low and high-intensity activity levels. Analyses included both descriptive and inferential statistics.

Descriptive statistics (means, standard deviations, percentages, and frequencies) summarized variable distributions by group (low and high-intensity exercise) and time point (pre and post intervention).

To evaluate the effects of the intervention over time, a repeated measures ANOVA was used to compare the pre and post-intervention scores within each group (low- and high-intensity exercise). This test allows us to examine the changes over time (pre vs. post) and to assess if there were significant differences within each group.

To assess intervention effects over time, repeated measures ANOVA was used to compare pre- and post-intervention scores within each group. A two-way repeated measures ANOVA evaluated differences between groups and tested for interaction effects between time (pre vs. post) and exercise intensity (low vs. high). Effect sizes were calculated using Cohen’s d (small = 0.2, medium = 0.5, large = 0.8). Analyses were conducted using SPSS v27, with significance set at *p* < 0.05.

## Results

### Flow of participants and demographic outcomes

**Fig. 2:**
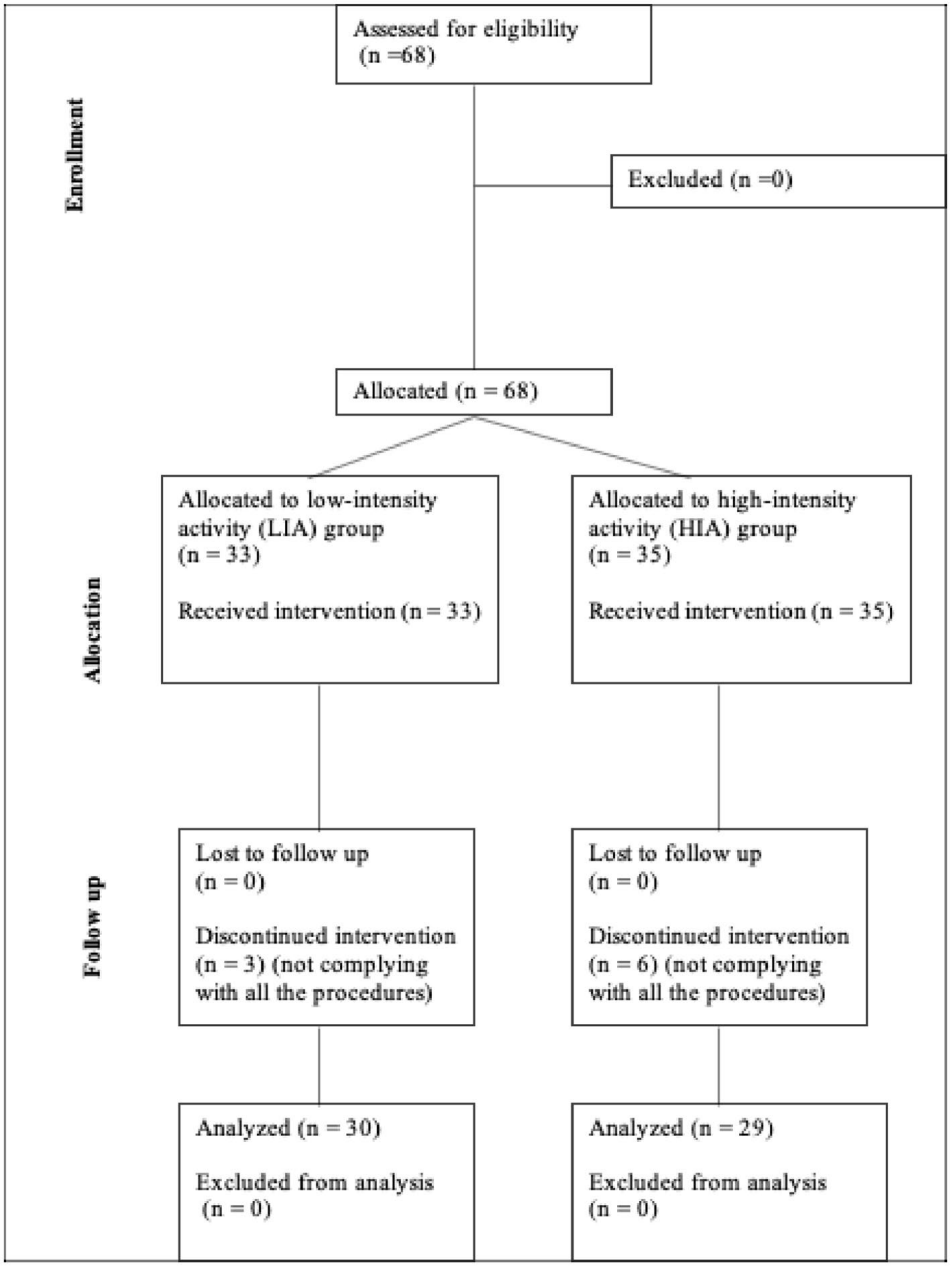
Flow chart of participants

A total of 68 subjects who expressed interest in participating in the study were evaluated for inclusion, with all of them meeting the inclusion criteria. Participants were divided into two groups: 33 engaged in low-intensity activity (LIA) and 35 in high-intensity activity (HIA). However, 9 participants (3 from the LIA group and 6 from the HIA group) did not complete the educational course intervention, and therefore, their data were not analyzed. This process is illustrated in Fig. 2.

Demographic outcomes are presented in Table 1, showing that the mean age of participants was 25.5 (4.5) years, with no statistical differences (*p* > 0.05) between the groups. No statistical differences were observed in terms of height, weight, or body mass index.

**Table 1:** Results for PIKQ (prolapse and incontinence knowledge questionnaire)

	Group	Time	Mean (SD)	Pre-post (*p* value)	Effect size (*d* Cohen)	Between-group comparison (time × group)
PIKQ UI	Low intensity (*n* = 30)	Pre	10.03 (1.52)	0.105	0.306	0.109
		Post	10.50 (1.43)			
	High intensity (*n* = 29)	Pre	9.31 (1.69)	0.109	0.307	
		Post	9.83 (1.51)			
PIKQ POP	Low intensity	Pre	8.07 (2.42)	0.005*	0.553	< 0.001*
		Post	9.43 (1.94)			
	High intensity	Pre	6.93 (2.56)	< 0.001*	0.933	
		Post	9.55 (2.57)			
PIKQ Overall	Low intensity	Pre	18.10 (3.05)	< 0.001*	0.668	< 0.001*
		Post	19.33 (3.06)			
	High intensity	Pre	16.24 (3.74)	< 0.001*	0.825	
		Post	19.38 (3.88)			

### Effects of the intervention

#### Knowledge of the pelvic floor

Pelvic floor knowledge, including an understanding of UI and POP, was assessed using the PIKQ. Overall scores significantly increased following the intervention in both the LIA group (*p* = 0.001, Cohen’s *d* = 0.668) and the HIA group (*p* = 0.001, Cohen’s *d* = 0.825), indicating a moderate to large effect size. Specifically, the POP domain exhibited enhanced outcomes in both groups, also with a moderate to large effect size. Notably, the high-intensity activity group demonstrated significantly greater gains in both overall knowledge and POP-specific scores (*p* < 0.001) (Table 2).

**Table 2:** Results for the FSFI questionnaire (female sexual function index)

	Group	Time	Mean (SD)	Pre-post (*p* value)	Effect size (*d* Cohen)	Between-group comparison (time × group)
Domain 1: desire	Low intensity (*n* = 30)	Pre	4.12 (0.98)	0.046*	− 0.381	0.257
		Post	4.52 (0.97)			
	High intensity (*n* = 29)	Pre	3.89 (0.93)	0.396	− 0.160	
		Post	4.01 (0.95)			
Domain 2: arousal	Low intensity	Pre	5.17 (1.10)	0.027*	− 0.426	0.450
		Post	5.34 (1.11)			
	High intensity	Pre	4.82 (1.64)	0.791	− 0.050	
		Post	4.86 (1.78)			
Domain 3: lubrication	Low intensity	Pre	4.95 (1.33)	0.321	− 0.184	0.986
		Post	5.06 (1.18)			
	High intensity	Pre	4.73 (1.78)	0.547	− 0.113	
		Post	4.85 (1.39)			
Domain 4: orgasm	Low intensity	Pre	4.80 (1.38)	0.075	− 0.337	0.237
		Post	4.99 (1.27)			
	High intensity	Pre	4.37 (1.86)	0.33	− 0.416	
		Post	4.83 (1.66)			
Domain 5: satisfaction	Low intensity	Pre	5.17 (0.82)	0.062	− 0.354	0.867
		Post	5.44 (0.64)			
	High intensity	Pre	4.91 (1.41)	0.88	− 0.329	
		Post	5.14 (1.10)			
Domain 6: pain during intercourse	Low intensity	Pre	5.11 (0.82)	0.304	− 0.191	0.326
		Post	5.44 (0.65)			
	High intensity	Pre	4.68 (1.83)	0.049*	− 0.383	
		Post	5.05 (1.49)			
Overall score	Low intensity	Pre	29.23 (5.53)	< 0.001*	− 0.688	0.973
		Post	30.60 (4.87)			
	High intensity	Pre	27.21 (8.54)	0.088	− 0.320	
		Post	28.75 (7.10)			

#### Sexual function

Specifically, among women regularly engaged in low-intensity activities, the six-week online video educational program led to significant improvements with a moderate effect size in the overall score of the FSFI-19 Questionnaire (*p* < 0.001, *d* Cohen 0.688), as well as in the specific domains of desire (*p* = 0.046, *d* Cohen = 0.381) and arousal (*p* = 0.027, *d* Cohen = 0.426). No significant pre-post differences were found in the remaining FSFI domains (*p* > 0.05). In the group composed of women who perform high-intensity activity, a significant improvement was observed post-intervention in the FSFI domain related to pain during intercourse (*p* = 0.049, *d* Cohen = 0.383), but not in the rest of the domains.

No significant interaction effects were found between improved outcomes and group allocation (Table 3).

**Table 3:** Results for BREQ (behavioral regulation in exercise questionnaire)

	Group	Time	Mean (SD)	Pre-post (*p* value)	Effect size (*d* Cohen)	Between-group comparison (time × group)
Domain 1: amotivation	Low intensity (*n* = 30)	Pre	4.53 (1.19)	0.712	0.068	0.607
		Post	4.47 (1.22)			
	High intensity (*n* = 29)	Pre	4.83 (2.94)	0.693	0.074	
		Post	5.00 (3.23)			
Domain 2: external regulation	Low intensity	Pre	5.93 (2.70)	0.685	0.075	0.657
		Post	5.77 (2.30)			
	High intensity	Pre	5.93 (4.31)	0.453	0.141	
		Post	5.41 (3.02)			
Domain 3: introjected regulation	Low intensity	Pre	9.03 (3.20)	0.228	0.225	0.652
		Post	8.53 (2.86)			
	High intensity	Pre	8.52 (3.83)	0.884	0.037	
		Post	8.38 (3.53)			
Domain 4: identified regulation	Low intensity	Pre	17.33 (1.97)	0.181	0.250	0.287
		Post	16.80 (2.23)			
	High intensity	Pre	16.52 (4.07)	0.639	0.088	
		Post	16.86 (3.90)			
Domain 5: intrinsic regulation	Low intensity	Pre	17.70 (2.45)	0.667	0.077	0.928
		Post	17.53 (3.51)			
	High intensity	Pre	17.66 (4.65)	0.946	0.013	
		Post	17.59 (4.81)			
Overall score	Low intensity	Pre	54.53 (5.67)	0.334	0.175	0.862
		Post	53.65 (6.41)			
	High intensity	Pre	53.45 (11.57)	0.759	0.057	
		Post	52.90 (10.44)			

#### Motivation towards physical activity

BREQ-2 was used to assess motivation towards physical activity. Results indicated that the proposed intervention did not lead to changes in the levels of motivational regulation among the participants, as no significant improvements were observed in any of the domains (Table 4).

**Table 4:** Results for EQOL-5D

	Group	Time	Mean (SD)	Pre-post (p value)	Effect size (d Cohen)	Between-group comparison (time × group)
Mobility	Low intensity (*n* = 30)	Pre	3.00 (0)	n.e	n.e	n.e
		Post	3.00 (0)			
	High intensity (*n* = 29)	Pre	3.00 (0)	n.e	n.e	
		Post	3.00 (0)			
Self-care	Low intensity	Pre	3.00 (0)	n.e	n.e	n.e
		Post	3.00 (0)			
	High intensity	Pre	3.00 (0)	n.e	n.e	
		Post	3.00 (0)			
Daily activities	Low intensity	Pre	2.93 (0.25)	0.326		0.182
		Post	2.97 (0.18)			
	High intensity	Pre	3 (0)	0.336		
		Post	2.93 (0.31)			
Pain	Low intensity	Pre	2.83 (0.38)	0.161		0.978
		Post	2.77 (0.43)			
	High intensity	Pre	2.83 (0.38)	0.326		
		Post	2.76 (0.43)			
Anxiety/Depression	Low intensity	Pre	2.77 (0.43)	0.031*		0.072
		Post	2.57 (0.56)			
	High intensity	Pre	2.66 (0.48)	0.712		
		Post	2.69 (0.54)			
Overall score	Low intensity	Pre	8.13 (1.63)	0.286		0.275
		Post	8.43 (0.93)			
	High intensity	Pre	8.48 (0.95)	0.712		
		Post	8.41 (1.05)			

#### General health status

The EQOL-5D assessed general health status, revealing only a significant improvement after the intervention for the anxiety and depression question among women in the low-intensity group (*p* = 0.031) (Table 5).

## Discussion

The present study aimed to analyze the effects of an online educational program on pelvic floor health knowledge among women engaged in low- and high-intensity training, in terms of sexual function, quality of life, physical activity, and satisfaction. The findings suggest that an online educational intervention, consisting of six educational videos, was effective in enhancing certain aspects of sexual function and quality of life among women participating in both low and high-intensity training. However, no improvements were observed regarding physical activity. Likewise, the effects were very similar between the two physical activity level groups, with only different responses found in certain aspects related to pelvic floor knowledge. Hence, given that this intervention is delivered online—offering flexible access to content and requiring minimal resources—its potential applications are promising. Future research should explore the optimal parameters and characteristics required to maximize improvements in women’s pelvic floor health knowledge.

The ultimate goal of the proposed intervention was to enhance education on pelvic floor knowledge as a preventive strategy for pelvic floor disorders among female athletes. By addressing a critical gap in the management of these conditions, the program targets a population that often lacks adequate information [27]. Indeed, insufficient information about pelvic floor disorders remains a widespread concern among women who are either affected by or at high risk of developing these conditions [15]. This knowledge gap is further influenced by socioeconomic and cultural factors, which can limit access to targeted educational resources [27]. Evidence from different health contexts, including pelvic floor disorders, suggests that educational programs can significantly improve clinical outcomes [12, 28]. Enhanced understanding of the pelvic floor is associated with greater corporal awareness, which facilitates the understanding of medical guidance and adherence to treatment recommendations provided by healthcare professionals [12]. Additionally, improved education has been associated with psychological benefits, such as reduced anxiety, increased motivation, and greater patient satisfaction [29]. Despite these promising outcomes, research specifically focused on female athletes remains limited. Notably, some studies indicate that a significant proportion of these women declare difficulties in correctly activating the pelvic floor muscles, highlighting the need for tailored educational interventions [30, 31].

eHealth interventions, defined as those delivered or enhanced through the internet and related technologies [32], are proposed as a potentially feasible tool to provide educational content among this population [33]. These programs enable users to access content and follow medical recommendations remotely and asynchronously, thereby helping to overcome one of the main barriers to educational interventions: the lack of time and accessibility [34]. Indeed, factors such as conflicting work and personal schedules, logistical challenges, and financial constraints are frequently reported as obstacles that prevent individuals from attending educational sessions or structured training programs focused on pelvic floor awareness [12, 35]. A review by Xu et al. [21] identified several eHealth modalities used in the literature to prevent or treat pelvic floor disorders, including mobile apps, internet websites, audiovisual content, and telephone sessions, which have been shown to improve self-efficacy. The findings from this systematic review with meta-analysis are consistent with our results, which suggest that medical content delivered via online platforms may increase self-efficacy, particularly in areas related to sexual function and pelvic floor knowledge. The obtained outcomes also align with those reported by Vico-Moreno et al. [36], who found that an online workshop including 3D anatomical models and guided proprioceptive exercises improves sexual function among track and field athletes.

Likewise, in the present study, knowledge about sexual function improved following the implementation of the educational program. This finding aligns with previous research indicating that awareness of the role of pelvic floor muscles in enhancing sexual function is generally low [37]. Indeed, more than half of the women evaluated in the study by Bosch-Donate et al. [38] believed it was normal to experience pain or discomfort during sexual activities. Such results underscore the need to address the persistent taboo surrounding dyspareunia by raising awareness, providing education, and promoting knowledge and self-care. Educational programs aimed at changing beliefs about pelvic pain are particularly useful in young adults, a population in which pelvic floor disorders are relatively common and responsiveness to educational strategies appears to be greater [39]. This supports the potential impact of the current intervention, which targeted physically active women aged 20 to 30. In contrast, de Andrade et al. [40] reported no improvements in sexual function after a four-week educational program delivered to women without a diagnosis of urinary incontinence. Although the content of that intervention was similar to ours, it was shorter in duration and delivered through in-person group sessions. In this regard, video-based content offers distinct advantages: it is more visually and dynamically engaging, facilitates greater user involvement, and allows for repeated viewing to enhance comprehension.

In addition, regarding the knowledge of UI and POP, it was observed that the intervention specifically improved POP, but not UI knowledge. A recent study published in 2025 reported similar findings [36], indicating no improvement regarding UI knowledge after an educational program. This study suggests that, in recent years, there has been an increase in awareness campaigns and programs related to UI. These efforts have contributed to higher baseline knowledge in the general population. Moreover, supporting this idea, studies from 2018 already showed improved levels of UI knowledge following pelvic floor education [40]. In contrast, educational interventions specifically addressing POP have been less frequent, resulting in persistently lower levels of awareness. This disparity may have also been present in our sample, which could explain the more pronounced improvements observed in POP-related knowledge [41].

Although different risks for developing IU and POP have been attributed depending on the intensity and impact of training, no previous studies have evaluated whether educational programs were more effective in those with greater risk (high-intensity training). The present study found similar improvements for both groups, with only minor superior effects in POP knowledge favoring the high-intensity group. Hence, the proposed educational program is feasible and potentially effective for all active women, regardless of being engaged in low or high-intensity activities.

### Limitations

The present study has several limitations that should be acknowledged. First, the study design did not include a control group for comparison. Therefore, although the feasibility of the proposed program to improve sexual function and knowledge has been demonstrated, its effectiveness should be further evaluated by comparing it with other interventions that incorporate educational or practical components related to pelvic floor health. Second, sexual function is a complex phenomenon influenced by multiple physical and psychological factors, many of which were not assessed in this study. Finally, the online format of the intervention entails an inherent limitation: participants only reported whether they had viewed the video. However, their ability to retain the information provided was not assessed, which may have influenced the results obtained.

## Conclusions

Educational pills on pelvic floor health, delivered through online videos, show promising results in improving sexual function and pelvic floor disorder knowledge among physically active women engaged in both low- and high-intensity training. Given the significant risk of pelvic floor disorders among young women who engage in physical activity and the demonstrated need to enhance knowledge in this area, further exploration of educational interventions is justified. Future studies should investigate the optimal parameters and characteristics needed to maximize improvements in women’s knowledge and awareness of pelvic floor health.

## Data Availability

No datasets were generated or analyzed during the current study.

## Appendix 1 List, summary and link of the six videos comprising the educational course

**Table:**

Title video	Summary of content	Link
What is the pelvic floor?	The main anatomical regions of the pelvic-perineal area are shown using a model, each of their functions (stability, posture, breathing, and urinary continence) is explained, and practical guidelines are provided to perform a voluntary pelvic floor contraction	https://youtu.be/nmaw9py5AMI?feature=shared
What is urinary stress incontinence?	The differences between the various types of incontinence are highlighted, along with the concept of stress urinary incontinence (SUI) and its relationship to certain everyday and sporting gestures	https://youtu.be/owq7lL37Zac?feature=shared
Stress urinary incontinence and prevention	A recap of the previous explanation is provided, addressing the risk factors that can exacerbate pelvic floor dysfunctions, lifestyle and habits, SUI symptoms, breathing concerning SUI, and general guidelines for applying preventive measures in high-pressure situations	https://youtu.be/ItnJmI5RFUo?feature=shared
Practical awareness of the pelvic floor	An appropriate pelvic floor contraction is demonstrated practically, highlighting typical mistakes in execution. It is practiced in positions that require increasing control, progressing towards a posture less favored by gravity but more functional	https://youtu.be/vEWbJXXMGsk?feature=shared
Prevention of stress urinary incontinence and pelvic floor training (I)	Preventive and treatment techniques supported by current literature are explained, focusing on strengthening the pelvic floor muscles and providing examples of training programs	https://youtu.be/SjH1xZwncvA?feature=shared
Prevention of stress urinary incontinence and pelvic floor training (II)	Useful elements for pelvic floor training and its progression are explored (vaginal cones and pessaries). The importance of the core and its activation, as well as stretching and muscle relaxation as preventive methods alongside strength training programs, is also emphasized	https://youtu.be/qRlZTRBlhYI?feature=shared
